# Synchronous Occurrence of Papillary Carcinoma in the Thyroid Gland and Thyroglossal Duct in an Adolescent with Congenital Hypothyroidism

**DOI:** 10.4274/jcrpe.477

**Published:** 2012-03-08

**Authors:** Zeynep Şıklar, Merih Berberoğlu, Aydın Yağmurlu, Bülent Hacıhamdioğlu, Şenay Savaş Erdeve, Suat Fitöz, Metin Kır, Gönül Öçal

**Affiliations:** 1 Ankara University Faculty of Medicine, Department of Pediatrics Endocrinology, Ankara, Turkey; 2 Ankara University Faculty of Medicine, Department of Pediatric Surgery, Ankara, Turkey; 3 Ankara University Faculty of Medicine, Department of Pediatric Radiology, Ankara, Turkey; 4 Ankara University Faculty of Medicine, Department of Nuclear Medicine, Ankara, Turkey; +90 312 595 67 91+90 312 319 14 40zeynepsklr@gmail.com

**Keywords:** Congenital, hypothyroidism, differentiated thyroid carcinoma, synchronous, thyroglossal duct carcinoma

## Abstract

Thyroid carcinoma (TC) combined with congenital hypothyroidism is rare. The synchronous occurrence of these two conditions is even rarer. We describe a patient with congenital hypothyroidism in whom hyperthyroglobulinemia and nodules developed despite adequate replacement therapy. Papillary TC was detected at age 19 years. Postoperative diagnostic scintigraphy showed increased uptake in the thyroglossal duct region. Repetitive imaging of the thyroid gland can be useful in the early detection of TC in patients with congenital hypothyroidism. Moreover, this rare situation can be complicated by a synchronous thyroglossal duct carcinoma. Thyroglossal duct carcinoma can be detected if diagnostic scintigraphy is performed after total thyroidectomy.

**Conflict of interest:**None declared.

## INTRODUCTION

Differentiated thyroid carcinoma (TC) represents 0.4%-3% of all childhood malignancies (1). The development of TC combined with congenital hypothyroidism is a rare situation (2,3). A few cases of TC arising from congenital goitre . have been reported until now (4,5,6).

An ectopic thyroid gland in a thyroglossal duct remnant may be present in infants with congenital hypothyroidism (7). Another developmental anomaly which can present with congenital hypothyroidism is a thyroglossal duct cyst. Approximately 1% of all thyroglossal duct cysts lead to TC, but synchronous development of thyroglossal cyst carcinoma and TC is extremely rare (8,9,10).

In this report, we describe a patient presenting with congenital hypothyroidism in the neonatal period and who subsequently developed synchronous thyroglossal cyst carcinoma and TC. 

## CASE REPORT

A 13-day-old male infant presented to our clinic with goitre and hypoactivity. He was the first child of parents who were first-degree relatives. The father had undergone thyroidectomy for multinodular goitre. A state of primary congenital hypothyroidism was detected in our patient. His total thyroxine (T4) level was 1.6 μg/dL (N: 4.5-12), his total triiodothyronine level (T3) 0.6 ng/mL (N: 0.55-2.5), and his thyrotropin (TSH) level was 84 mIU/mL (N:0.5-5). T4 replacement therapy was initiated. In the first 3 years of follow-up, despite treatment and normal growth and development pattern, the patient continued to have persistently high TSH levels (mean TSH: 13 mIU/mL), despite upper-normal levels of free T4 (fT4). These high levels persisted for almost 9 years, but gradually decreased afterwards. At age 9 years, TSH: level was 3 mIU/mL. fT4, free T3 (fT3) and TSH levels remained within normal limits thereafter (Table 1).

**Figure 1:**
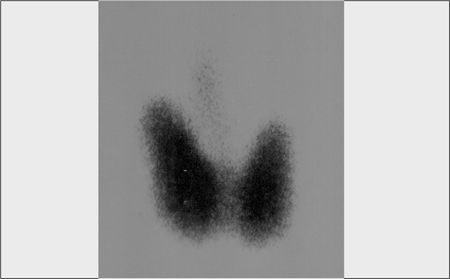
Thyroid scintigraphy before diagnosis of thyroid carcinoma

When he was 13.4 years of age, the patient developed a goitre and hyperthyroglobulinemia. Thyroid ultrasound demonstrated a 4-mm solid nodule in the right lobe. Fine-needle aspiration (FNA) biopsy did not reveal any pathology. Thyroid scintigraphy showed a diffuse hyperplastic thyroid gland (Figure 1). Thyroid autoantibodies were negative and urinary iodine level was normal.

During follow-up, an increase in thyroid volume, hyperthyroglobulinemia (with fluctuating levels reaching up to 1100 ng/mL), and new nodule formation were noted. A second FNA biopsy performed at age 16 years showed no malignancy. One year later, upon detection of a cold nodule measuring 9mm in diameter in the right lobe, a right lobectomy was performed. Histological examination revealed follicular adenoma. Following the right lobectomy, thyroglobulin (Tg) levels showed a decrease initially, but started to increase one year later together with a gradual increase in the size of nodules in the left lobe, which led to a decision for total thyroidectomy with central neck lymph node dissection. Histological examination revealed findings compatible with follicular variant of papillary TC; tumor size was 6 mm. 

**Figure 2:**
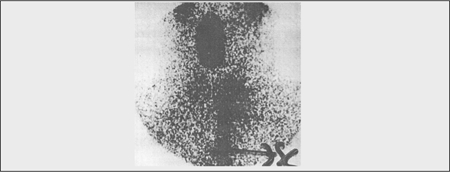
Low-dose I-131 scintigraphy after thyroidectomy

Although not detected on preoperative scintigraphy, postoperative diagnostic scintigraphy with low dose of I-131 (2 mCi) showed increased uptake in the thyroglossal region (Figure 2). Surgical exploration for thyroglossal remnants revealed a presence of a follicular variant of papillary TC which had developed synchronously. Levothyroxine (LT4) replacement therapy and radioiodine ablation of the possible remnant thyroid tissue were started. At this time, we learnt that the patient’s sister had been diagnosed as a case of euthyroid diffuse goitre and that she also had developed a follicular adenoma in the follow-up.

## DISCUSSION

The case presented here had goitrous congenital hypothyroidism and developed TC in his adolescent years. Only a few cases of TC arising from dyshormonogenetic goitre of the thyroid gland have been reported to date (11,12). In our patient, despite adequate replacement therapy, the TSH level had not been suppressed completely until he was 9 years old. High TSH levels are believed to induce the thyroid gland, leading to changes such as nodule formation. It has been suggested that constant and prolonged stimulation by TSH may lead to occurrence of malignant thyroid cells (2). It has also been suggested that combined with dyshormonogenetic congenital hypothyroidism, ineffective treatment of congenital hypothyroidism and occurrence of high TSH levels from time to time may be responsible for pathophysiological mechanisms leading to the development of TC (11). Malignant transformation can occur if elevated TSH levels are sustained for a prolonged period of time (2).

Our patient was compliant to therapy, with almost no reported instances of missed LT4 dose. In the first years of LT4 therapy, high-normal fT4 levels were accompanied by mild TSH increments, a finding resembling a state of mild resistance to TSH suppression. The patient developed TC 10 years after the normalization of TSH levels. Thus, we suggest that the high TSH levels noted in the patient’s first 9 years of life could have contributed to TC development at age 19 years. We believe that the high TSH levels appear to be a probable etiological factor for TC in this patient.

There was also a tendency to formation of thyroid nodules in our patient’s family. Some genetic defects are known to facilitate carcinoma development in patients with congenital hypothyroidism. It was reported that metastatic TC can arise from congenital goitre due to Tg or thyroperoxidase gene mutations (2,5). We were not able to investigate thyroid-associated gene mutations in this patient. However, unlike the findings in our patient, almost no Tg is detected in patients with Tg gene mutations. Fluctuating increased Tg levels found in our patient warn us about the possibility of elevated Tg levels being a key factor for TC development.

In our patient, following the surgical intervention to the thyroid gland, a low-dose radioiodine scanning showed minimal uptake in the thyroid bed, but increased uptake in the thyroglossal duct region. Interestingly, this region could not be visualized scintigraphically before thyroidectomy.

Iodine ablation after thyroidectomy and postablative whole-body scintigraphy are important components of the management of TC in children and adolescents. Before high-dose radioiodine ablation, one should be sure that no extra thyroidal tissue is left. The success of radioiodine ablation depends on the amount of thyroid tissue. If the amount of remnant tissue is high, most of the radioiodine dose will be trapped by this tissue and the effectiveness of ablation will be decreased. Also, distant metastases cannot be detected. If radioiodine ablation is applied before performing diagnostic low-dose I-131 scintigraphy, almost all of the ablation dose will be trapped by thyroglossal duct remnants and it will not be possible to detect presence of distant metastases.

In this patient, we detected synchronous development of differentiated papillary carcinoma in the thyroid gland and thyroglossal duct. First, thyroid gland carcinoma, and subsequently thyroglossal duct carcinoma were diagnosed. Functional follicular thyroid cells can be found in thyroglossal duct remnants (7). TC can arise in thyroglossal duct cysts rarely, with fewer than 200 cases reported. The incidence was reported as 1.3% of all thyroglossal duct cysts. About 1% of patients with thyroglossal duct cysts were treated for papillary TC arising in thyroglossal duct cysts (12), and in most of these patients, TC was diagnosed in the fourth decade of life (13). The largest series was reported from France, including 18 cases aged from 27 to 68 years (12). In children, TC originating from thyroglossal cyst has been reported in fewer than ten cases (13,14). However, the synchronous occurrence of TC and thyroglossal duct carcinoma is reported to be extremely rare (13). More than half of the patients in these series showed concomitant microcarcinoma (most of them smaller than 1 mm in diameter) in the thyroid gland (12).

The findings in our patient point out the importance of investigating the possible presence of TC in patients with thyroglossal duct carcinoma, and vice versa.

In conclusion, we would like to stress the importance of repetitive imaging of the thyroid gland in patients with congenital hypothyroidism, especially in those with high Tg levels and nodule formation. Thyroglossal duct carcinoma can develop in patients with congenital hypothyroidism. Moreover, this rare situation can be complicated by synchronous TC. Thyroglossal duct carcinoma can remain undiagnosed if diagnostic I-131 scintigraphy is not done after total thyroidectomy. 

## Figures and Tables

**Table 1:**
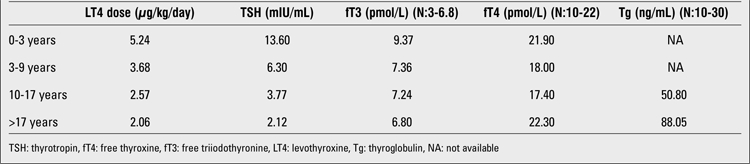
Mean LT4 doses and mean TSH, fT4, fT3 levels of the patient during follow-up
